# C-Reactive Protein and Genetic Variants and Cognitive Decline in Old Age: The PROSPER Study

**DOI:** 10.1371/journal.pone.0023890

**Published:** 2011-09-07

**Authors:** Simon P. Mooijaart, Naveed Sattar, Stella Trompet, Eliana Polisecki, Anton J. M. de Craen, Ernst J. Schaefer, Sabine E. Jahn, Thomas van Himbergen, Paul Welsh, Ian Ford, David J. Stott, Rudi G. J. Westendorp, on behalf of The PROSPER Study Group

**Affiliations:** 1 Department of Gerontology and Geriatrics, Leiden University Medical Center, Leiden, The Netherlands; 2 BHF Glasgow Cardiovascular Research Centre, University of Glasgow, Glasgow, Scotland; 3 Department of Cardiology, Leiden University Medical Center, Leiden, The Netherlands; 4 Tufts University, Boston, Massachusetts, United States of America; 5 Division of Cardiovascular and Medical Sciences, University of Glasgow, Glasgow, Scotland; 6 Robertson Centre for Biostatistics, University of Glasgow, Glasgow, Scotland; 7 Department of Geriatric Medicine, University of Glasgow, Glasgow, Scotland; 8 Netherlands Consortium for Healthy Aging, Leiden, The Netherlands; Institut National de la Santé et de la Recherche Médicale (INSERM), France

## Abstract

**Background:**

Plasma concentrations of C-reactive protein (CRP), a marker of chronic inflammation, have been associated with cognitive impairment in old age. However, it is unknown whether CRP is causally linked to cognitive decline.

**Methods and Findings:**

Within the Prospective Study of Pravastatin in the Elderly at Risk (PROSPER) trial, with 5680 participants with a mean age of 75 years, we examined associations of CRP levels and its genetic determinants with cognitive performance and decline over 3.2 years mean follow-up. Higher plasma CRP concentrations were associated with poorer baseline performance on the Stroop test (P = 0.001) and Letter Digit Tests (P<0.001), but not with the immediate and delayed Picture Learning Test (PLT; both P>0.5). In the prospective analyses, higher CRP concentrations associated with increased rate of decline in the immediate PLT (P = 0.016), but not in other cognitive tests (all p>0.11). Adjustment for prevalent cardiovascular risk factors and disease did not change the baseline associations nor associations with cognitive decline during follow-up. Four haplotypes of CRP were used and, compared to the common haplotype, carrierships associated strongly with levels of CRP (all P<0.007). In comparison to strong associations of apolipoprotein E with cognitive measures, associations of CRP haplotypes with such measures were inconsistent.

**Conclusion:**

Plasma CRP concentrations associate with cognitive performance in part through pathways independent of (risk factors for) cardiovascular disease. However, lifelong exposure to higher CRP levels does not associate with poorer cognitive performance in old age. The current data weaken the argument for a causal role of CRP in cognitive performance, but further study is warranted to draw definitive conclusions.

## Introduction

Plasma concentrations of C-reactive protein (CRP), a marker of chronic inflammation, have been associated with numerous clinical conditions, including cognitive decline in old age. However, although some epidemiological studies indeed suggest an association of high concentrations of CRP with risk of dementia or cognitive decline [1]–[6], the data are not consistent [7], [8]. A meta analysis [9] suggested an association with cognitive impairment, but to date causality is uncertain.

Investigation of mechanisms linking CRP to cognitive decline in humans is hampered by the inability to distinguish cause from effect in conventional association studies. Whether CRP directly causes cognitive decline in the brain is not well examined. Speculatively, CRP may indirectly lead to cognitive impairment via promoting vascular disease i.e. causing stroke and transient ischemic attacks (TIAs), although recent genetic studies go against CRP as a causal agent in vascular disease [10]. In another hypothesis CRP is produced as a result of the inflammatory process linked to a plethora of lifestyle factors (e.g. obesity, smoking, poor diet, lack of activity etc.) and is therefore simply a by-product linked to adverse lifestyle. So far, only few studies have considered all such possibilities or had the ability to take into account cardiovascular disease, risk factors and genetic determinants of CRP as means of testing causality.

Within the Prospective Study of Pravastatin in the Elderly at Risk (PROSPER) trial - a trial of pravastatin versus placebo among 5804 men and women, who were 70 to 82 years of age [11] - we associated plasma CRP concentrations with cognitive decline and investigated to what extent cardiovascular risk factors explain this association. Second, we investigated the effects of genetically determined elevated CRP concentrations as a proxy for lifelong higher CRP concentrations on risk of cognitive decline in old age, and compared such associations to known effects of apolipoprotein E (APOE) on cognitive measures as an internal “positive” control.

## Methods

### Study design and participants

PROSPER (Prospective Study of Pravastatin in the Elderly at Risk) was a trial of statin use in preventing coronary and cerebrovascular events in older subjects either with a previous history of vascular disease or at high risk of an event due to a history of smoking, hypertension or diabetes [11]. Subjects aged 70–82 years were recruited in Scotland, Ireland and the Netherlands and randomized to receive 40 mg pravastatin or placebo daily. There were restrictions on entry: plasma cholesterol had to be in the range 4.0–9.0 mmol/l and triglyceride <6.0 mmol/l. A cut off score of 24 points or more (out of 30) on the Mini Mental State Examination (MMSE) at baseline was used as an inclusion criterion to eliminate those with poor cognitive function at baseline. Statin use did not affect cognitive decline during the study period [12]. The study was approved by the institutional ethics review boards of centres of Cork University (Ireland), Glasgow University (Scotland) and Leiden Univeristy Medical Center (the Netherlands) and all participants gave written informed consent.

### Measurements

Plasma concentrations of cholesterol, triglyceride, low density lipoprotein (LDL) cholesterol and high density lipoprotein (HDL) cholesterol were measured twice at fasting visits during the placebo run-in phase in a central laboratory which was standardised through the Center for Disease Control network. Apolipoprotein E phenotype was determined on plasma samples by Western blotting following the method of Havekes et al [13]. Subjects were classified according to the presence of the E_2_, E_3_ or E_4_ bands on gel blots. CRP was measured on stored (at −80°C) and previously unthawed samples by automated particle-enhanced immunoturbidimetric assay (Roche, UK). The method has inter and intra-assay coefficient of variation of <3%. Our laboratory participates in a national external quality control for high sensitivity CRP. All samples were processed by technicians blinded to the identity of samples. CRP measurements were available for 5680 participants.

### Prevalent disease

Diabetes at baseline was defined by self-reported history, a fasting blood glucose measurement of ≥7.0 mmol/l, or in the 5% of subjects that did not fast, a non-fasting blood glucose measurement of ≥11.1 mmol/l, or self-reported use of anti-diabetic medications (any oral hypoglycaemic or insulin). Hypertension at baseline was defined by self-reported history; blood pressure was measured at baseline. Smoking was defined as current smoking at baseline. History of cardiovascular disease (myocardial infarction, stroke/TIA or vascular) was defined as self-reported at baseline. Level of education was assessed by the age the participant left school.

### Genotyping

Three single nucleotide polymorphisms (SNPs) in the *CRP* gene were selected (table 1) that have previously been associated with concentrations of CRP. Genotyping was carried out using TaqMan® SNPs genotyping assays (Applied Biosystems, Foster City, CA). The endpoint was read after PCR amplification using an Applied Biosystems 7900 HT Sequence Detection System. Genotypes with quality scores below the 95% were repeated and 5% blinded replicates for genotype determinations were performed. All three polymorphisms were in Hardy-Weinberg equilibrium (all *P*>0.17) and were available for all 5680 participants.

**Table 1:** Selected polymorphisms in the CRP gene.

rs-number	Position in gene	Coding	Alleles	Minor allele frequency
rs1417938	Intron 1	No	T/A	0.297
rs1800947	Codon 184	Synonymous	G/C	0.060
rs1205	3′UTR	No	C/T	0.330

**Minor alleles are underlined.**

### Cognitive test battery

A detailed description of the cognitive tests used in the study has been published earlier [14]. The Mini Mental State Examination (MMSE) is used widely to screen for cognitive impairment. Memory was tested using the Picture-Word Recall test based on the Groningen-Fifteen Words test [15]. This measures recall, both immediate and after 20 minutes, of 15 pictures. The outcome variable is the mean number of correctly recalled pictures over three immediate trials and number recalled after the delay. Attention and processing were assessed using the Stroop-Colour Word test and the Letter-Digit Coding test [14]. The former, in the key Part III of the test, presents colour names printed in incongruously coloured ink (e.g. the word ‘green’ printed in blue ink). Performance, timed in seconds to complete the test, measures the ability to discard the irrelevant name (green) in favour of the colour of the ink (blue). The latter asks the subject to fill in digits next to letters according to a key and the outcome is the number of correct entries in 60 seconds. Subjects were assessed with al tests twice (two weeks apart) at baseline to allow for any training effect. The results of the second test were taken as the starting estimate of cognitive function. All tests were repeated at 9, 18 and 30 months, and at final trial visit. The time point of this last measurement was different for the participants (at 36–48 months), therefore we performed the analyses with their individually varying time-point but report the results for the mean of these time points (at 42 months).

### Statistical analyses

The distribution of CRP was skewed and therefore a logarithmic transformation was used. Means and standard deviations were used to assess central tendency of normally distributed variables, whereas median and interquartile range were used for variables that were not normally distributed.

The program Haploview [16] was used to estimate the allele frequencies, test the consistency of the genotype frequencies at each SNP locus with Hardy–Weinberg equilibrium, and estimate and plot pairwise linkage disequilibrium (LD) between the SNPs examined. Haplotypes and haplotype frequencies were calculated using SNPHAP software (http://www-gene.cimr.cam.ac.uk/clayton/software, 2006). We used multiple imputation analysis to account for many haplotype probabilities per subject. This method has been described elsewhere in more detail [17]. Imputed haplotypes with frequencies below 5% are included in the models to ensure the use of all data. Associations with this combined group are however not reported, as inferences are hard to make from associates with this heterogeneous groupThe haplotype analysis approach used in this study assumes an additive effect of the haplotypes, and details of this approach have been described elsewhere [18].

To assess the relation between CRP concentrations, CRP haplotypes and cognitive function, we used linear mixed models. Mixed models use all available data during multiple visits during follow up, can properly account for correlation between repeated measurements, and can handle missing data more appropriately than traditional models. They allow for the use of time independent and time dependent covariates [19].

Baseline cognitive performance per tertile of CRP concentration was calculated using a linear regression model. The rate of cognitive decline during follow-up dependent on CRP tertile (‘accelerated cognitive decline’) was calculated with the interaction term between tertile of CRP with study visit in the linear mixed model, assessing whether cognitive decline is steeper in any of the tertiles.

To assess the relationship between *CRP* haplotypes with cognitive performance we used linear mixed models in a different way. For each cognitive test ‘mean difference over the study period’ was calculated between haplotypes of CRP, comparing the mean cognitive performance at all study visits per haplotype of CRP. Multiple imputation was used to account for haplotype probabilities. The model assumes that the effect of CRP genotypes on cognition exists throughout the lifetime, with a resulting difference in cognitive performance during the entire study period.

All models were adjusted for age, sex and country of inclusion. The baseline association of CRP concentrations and cognition was additionally adjusted for level of education and APOE genotype. In the prospective analyses of the association of CRP concentrations with the rate of cognitive decline we additionally adjusted for study allocation (pravastatin or placebo). To assess the relationship with classical risk factors for atherosclerosis, models additionally included cardiovascular risk factors history of diabetes, hypertension, myocardial infarction, stroke, TIA or vascular disease, LDL- and HDL-cholesterol, triglycerides, systolic and diastolic blood pressure. The associations of CRP haplotypes with cognition were adjusted for age, sex, country of inclusion, study allocation (pravastatin or placebo) and APOE genotype.

All analyses were performed using SPSS version 16.0.

## Results

### Baseline characteristics

Baseline characteristics of study participants have been described in detail elsewhere [11] In short, the present analysis included 5680 participants of which 2936 (52%) were female. Mean age at baseline was 75.3 years (range 70–82). Mean CRP concentration was 3.1 mg/L.

### C-reactive protein concentration and cognitive performance

At baseline, higher concentrations of CRP were associated with worse performance on the Stroop and Letter Digit coding Test (LDT) (table 2). No such association was observed with the immediate and delayed Picture Learning Test (PLTi and PLTd). The observed associations with LDT and Stroop were independent of age, gender, level of education, country, treatment allocation and APOE genotype as the model was adjusted for these factors. Additional adjustment for a history of cardiovascular disease as well as classical cardiovascular risk factors did not affect these associations. During follow-up higher concentrations of CRP associated with an increased rate of cognitive decline measured only with the immediate PLT (*P* = 0.016), but not in the other cognitive tests (table 2). Again, adjustment for cardiovascular risk factors did not affect the association.

**Table 2:** Association of circulating concentrations of CRP with cognitive test performance at baseline.

Cognitive test, mean (95% CI)		Tertile of CRP concentration			P for trend	
		Low	Intermediate	High	basic	adjusted
		<2.0 mg/L	2.0–4.8 mg/L	>4.8 mg/L	model*	model†
		n = 1893	n = 1896	n = 1891		
Stroop (seconds to perform task)	Baseline score	65.3 (63.5; 67.2)	66.3 (64.4;68.2)	67.6 (65.7;69.5)	0.001	0.007
	Annual change	+0.78 (+0.56;+1.00)	+0.48 (+0.26;0.70)	+0.77 (+0.52;+1.02	0.698	0.697
LDT (letters coded)	Baseline score	23.8 (23.3;24.3)	23.3 (22.8;23.8)	22.9 (22.4;23.4)	<0.001	<0.001
	Annual change	−0.44 (−0.49;−0.39)	−0.28 (−0.33;−0.24)	−0.36 (−0.42;−0.31)	0.167	0.173
PLTi (pictures recalled)	Baseline score	9.4 (9.2–9.5)	9.4 (9.3;9.6)	9.4 (9.3;9.5)	0.577	0.461
	Annual change	−0.01 (−0.03;+0.01)	−0.01 (−0.03;+0.01)	−0.05 (−0.07;−0.02)	0.016	0.016
PLTd (pictures recalled)	Baseline score	10.2 (10.0;10.4)	10.3 (10.1;10.5)	10.2 (10.0;10.4)	0.604	0.909
	Annual change	−0.07 (−0.10;−0.04)	−0.07 (−0.10;−0.04)	−0.07 (−0.10;−0.04)	0.109	0.108

Mean scores on cognitive test battery were calculated at baseline using a linear regression model with tertiles of CRP as independent variable. Statistical significance of the trend was calculated using this model with CRP concentrations as continuous variable.

*Adjusted for gender, age at baseline, study site, level of education, treatment allocation (none at baseline), APOE genotype, and (if applicable) version of test used.

† Additionally adjusted for history of hypertension, diabetes, vascular disease, myocardial infarction, stroke or transient ischemic attack, body mass index, systolic and diastolic blood pressure, smoking, LDL- and HDL-cholesterol, triglycerides.

### CRP haplotypes And association with crp concentration and cognition

**Figure 1:**
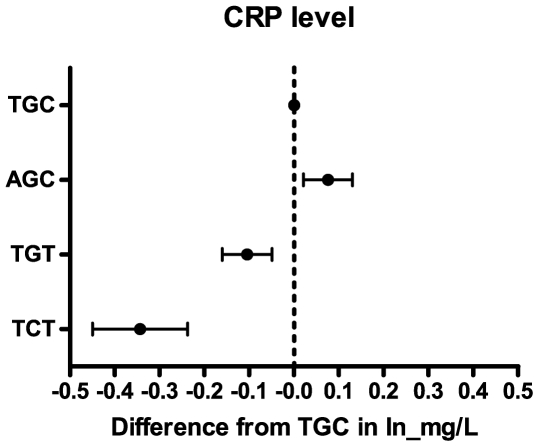
Association of *CRP* haplotypes with plasma concentrations of CRP. Data represent mean (dots) and 95% confidence interval (bars) of the difference in logarithmized CRP concentration of the different *CRP* haplotype as compared with the reference TGC haplotype.

**Figure 2:**
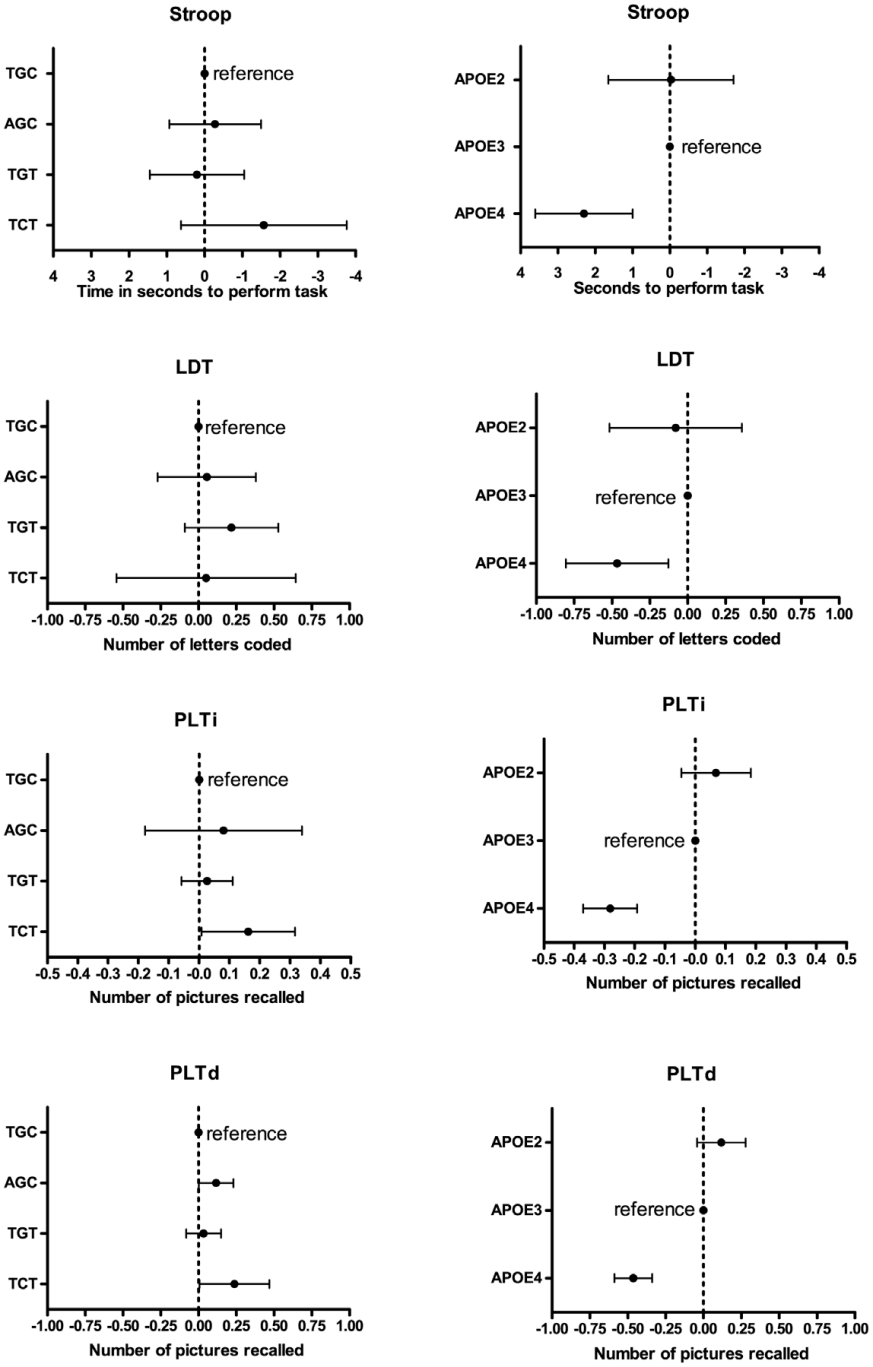
Association of *CRP* haplotypes and *APOE* epsilon 2/3/4 polymorphism with cognitive function. Association with cognitive performance on the repeated cognitive test battery for *CRP* haplotypes (A) and *APOE* haplotypes (B). Data represent mean and 95% confidence of the difference in test score for each allele compared to the reference TGC allele (*CRP*) or the epsilon 3 allele (*APOE*). Cognitive test scores are oriented such that left of the dotted line always represents worse performance, whereas right of the dotted line always represents better performance.

Allele frequencies of the three SNPs are listed in table 1. The three CRP polymorphisms constituted one block in tight linkage disequilibrium with four common haplotypes TGC (frequency 0.37) AGC (frequency 0.30), TGT (frequency 0.27) and TCT (frequency 0.06). Association of the haplotypes with plasma concentrations of CRP are shown in figure 1. Compared to the most frequent haplotype TGC, AGC associated with a higher plasma concentration of CRP (P = 0.007), whereas TGT and TCT associated with significantly lower concentrations of CRP (both P<0.001). The association of haplotypes of CRP with cognitive performance is shown in figure 2 (left panel). Compared to the most frequent haplotype TGC, AGC (and so higher CRP concentrations) was associated with better (not worse) performance on the PLTd (*P* = 0.044). The TGT haplotype did not associate with different cognitive performance on any test, compared to TGC. The TCT haplotype associated with better performance on both the delayed (*P* = 0.044) and immediate (*P* = 0.039) picture learning test. However, although highly statistically significant, the effect size of the association of *CRP* haplotypes with CRP concentrations is small. For instance, the TCT haplotype with the biggest difference with respect to CRP concentrations compared to the reference haplotype TGC had 0.35 logCRP lower levels (figure 1). For the cognitive test that associated the strongest with CRP concentrations (LDT), we calculated that this would lead to an expected effect of 0.12 points. From figure 2 it can be concluded that the estimates and especially the wide confidence intervals of the observed differences overlaps this expected estimate. We conclude that our study is underpowered to use the CRP haplotypes as an instrumental variable in a Mendelianrandomization approach. To give reader some sense of the magnitude of the association we used the *APOE* epsilon2/3/4 polymorphism as a positive control, to validate that using this approach we were able to find associations of common genotypes with cognitive performance using this cognitive test battery in this population. Figure 2 (right panel) shows an association of the APOE epsilon 4 allele with worse cognitive performance compared to the epsilon 3 allele, as measured with the Stroop (*P* = 0.001), LDT (*P* = 0.007), PLTi (*P*<0.001) and PLTd (*P*<0.001).

## Discussion

This study has two main findings. First, whereas there was substantial association of plasma CRP concentrations with cognitive performance in the cross-sectional analyses, the association was only minimal in the longitudinal analysis (which, given that tests are repeated within individuals, is perhaps a more rigorous test of association). In each case, observed associations were not affected by adjustment for cardiovascular risk factors or baseline vascular disease status. Second, although there was strong association between *CRP* haplotypes with CRP concentrations, the *CRP* genotype did not associate strongly (all P≥0.039) or consistently in the hypothesized direction with cognitive decline, in stark contrast to the observed more robust (all P≤0.007) and directionally consistent effects of apolipoprotein E (*APOE*) polymorphisms on cognition.

There are a number of explanations how circulating concentrations of CRP would associate with cognitive performance. There may be a direct causal effect of CRP inflicting damage locally in the brain. As CRP is not produced locally in the brain, this theory is dependent on the assumption that the blood-brain-barrier is permeable for CRP. Alternatively, CRP may indirectly influence cognition via potential effects on vascular disease. However, we found limited and inconsistent associations of haplotypes of *CRP* with cognitive function in a large population which has been exposed for over 70 years to germline determined (hence unconfounded, [20]) differential concentrations of plasma CRP. However, definitive conclusion cannot be drawn as power in our study was limited. A recent Mendelian Randomization study by Zacho [10] already reported no association of genetic variants in the *CRP* gene with cardiovascular disease. More recently, no association was observed of variants in the *CRP* gene with cognitive performance in old age in over four thousand individuals in four Scottish cohorts [21]. However, the four cohorts were heterogeneous in population selection, as were their measures of cognitive performance. These points leave room for speculation that this heterogeneity may have obscured an existing association. Furthermore, genetic association studies need replication. Our study now replicates in a large cohort the findings of Marioni. Furthermore, in our own study statins, that are known to reduce concentrations of CRP did not affect cognitive function, although we do not have repeated CRP measurements in our cohort. Taken together, these data currently do not support a role for CRP in cognitive decline, either directly causal or indirectly as a causative factor in vascular disease, but further study is necessary to draw definitive conclusions.

If CRP itself is not directly causal in cognitive decline, concentrations of CRP may be the read-out of mechanisms that causes cognitive decline. Again, cardiovascular disease and associated risk factors and lifestyle factors are the most likely candidates. Atherosclerosis may be regarded as a pro-inflammatory condition [22] and is also a consequence of cardiovascular risk factors such as diabetes mellitus, high blood pressure, and dyslipidaemia, which are all risk factors for cognitive decline [23] and are likewise associated with circulating concentrations of CRP [24]. We report here an association of concentrations of CRP with cognitive performance at baseline, and with an increased rate of cognitive decline during follow-up, at least in one cognitive domain. This association was however unaffected by adjustment for cardiovascular risk factors, suggesting that cardiovascular disease or risk factors, at least as measured, are not the simple explanations for the observed associations. Of course there may be residual confounding by unmeasured factors which raise CRP and also cause cognitive decline. We also cannot discount a reverse causal mechanism whereby an increased CRP is contributed to by cognitive decline; cognitive decline is associated with a poor nutritional status and increase in incident diseases, which in turn could lead to an increased plasma CRP concentration. Thus, although we see some associations of CRP with some domains of cognitive performance with decline, these observational data cannot establish causality.

Using known *CRP* polymorphisms, we noted a strong association between haplotypes of *CRP* with CRP concentration. The magnitude of these associations were, however, small and did not allow to perform a Mendelianrandomization analysis. We used the *APOE* epsilon 2/3/4 polymorphism as a positive control or benchmark, to illustrate that our approach is suitable to detect significant differences in cognitive performance. Interestingly, the *APOE* 2/3/4 alleles also associate with concentrations of CRP but we were carefully to adjust for *APOE* genotype in models linking CRP to cognitive measures. The epsilon 4 allele is associated with cognitive decline in many studies, including the one under study [25], thus providing some external validity of PROSPER findings. Based on our analysis, however, we cannot make definitive conclusions, which will have to come from studies large enough to perform a mendelianrandomization, and will likely have to involve a number of participants that is far greater than our cohort.

The present study adds to current understanding in two ways. First, we report on prospective follow-up of cognition in a cohort of over five thousand elderly, yielding a high power to measure the actual rate of cognitive decline within the individual. This approach is more robust to detect cognitive decline than cross-sectional association studies of CRP with cognition, as the latter studies are more vulnerable to confounding and individual variance in test performance. Second, this is the largest study we are aware to study the association of *CRP* genotypes with cognitive decline. These two aspects together substantially lessen support for CRP as causal agent in cognitive decline and as a consequence suggest a need for new thinking in this area of research. Besides larger studies as mentioned above, more research is warranted in the ‘upstream’ mechanisms/pathways causing CRP to rise concomitantly with cognitive decline, for example an examination of pro-inflammatory cytokines. We recognize, however, that our data require replication in other cohorts. Exclusion of elderly with an MMSE below 24 point at baseline, may have further reduced our power to detect changes in cognitive function. The observed associations between CRP concentrations and cognitive test scores do not allow for clinical utility as the effect sizes are small.

In conclusion, although CRP concentrations relate strongly to baseline executive function (Stroop and LDT) in the elderly, associations with annual change are less impressive and haplotypes of *CRP* that associate strongly with plasma CRP concentrations do not consistently associate with cognitive performance or decline in old age, in stark contrast to *APOE* genotype. The overall results argue against a causal role for CRP in cognitive performance or decline in older age, although more research is needed to provide definitive answers.
